# Crossing the Research to Quality Chasm: A Checklist for Researchers and Clinical Leadership Partners

**DOI:** 10.1007/s11606-017-4189-5

**Published:** 2017-10-02

**Authors:** Julie A. Schmittdiel, Richard W. Grant

**Affiliations:** Division of Research, Kaiser Permanente Northern California, Oakland, CA USA

The U.S. health care system faces a daunting set of challenges, including high costs, disparities in access, and significant gaps between evidence and practice.^1^ But despite these concerns, less than 0.1% of the over $3.2 trillion spent on health care in the U.S. goes toward research to improve how we *deliver* care.^1^^,^ 2

Delivery science research seeks to overcome barriers, leverage facilitators, and implement innovations to improve care.^2^ While partnerships between clinical and operations leaders and delivery science researchers can play a significant role in creating new evidence and quickly closing gaps between evidence and practice,^1^^,^ 3 these collaborations are often limited in both number and scope. To achieve the goal of creating a learning health system,^4^ researchers and health system leaders must work together to develop and evaluate new programs, learn from real-world experiments in health care delivery and policy, disseminate knowledge, and implement more effective and efficient care strategies (Fig. 1). However, successful cross-cutting collaborations can be challenging due to differing timelines, sources of financial support, career goals, and measures of success.^3^^,^ 5 Here we propose a “Collaboration Checklist” to help researchers and clinical leaders more successfully navigate and address key strategic issues when contemplating a partnership between research and operations.

**Figure 1:**
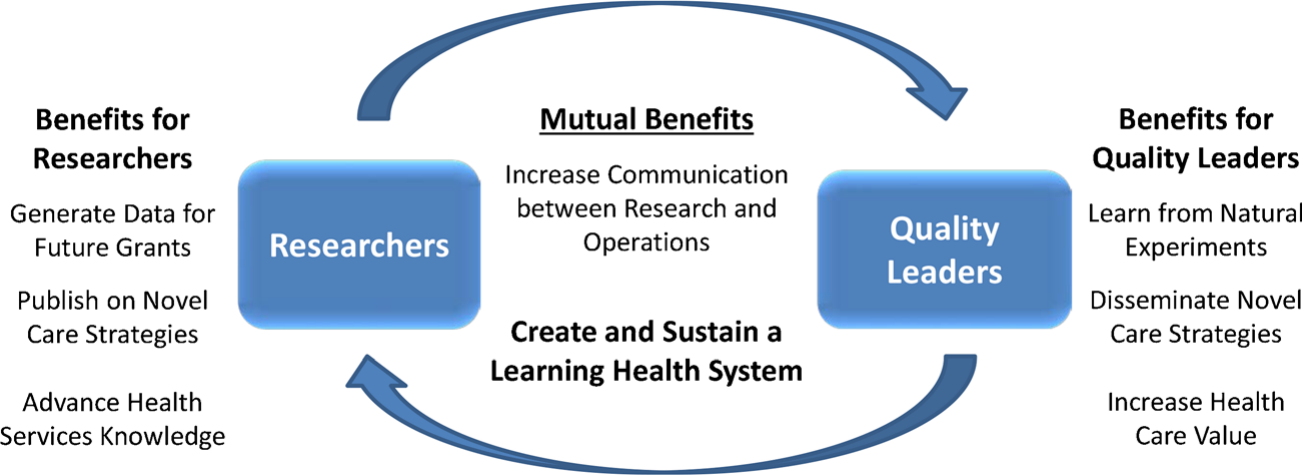
Benefits of research and clinical quality improvement partnerships.

## UNDERSTANDING THE STAKEHOLDER NEEDS

The first step is for potential collaborators to gain a clear understanding of exactly *what* questions need to be answered and *why* they are being asked. Before collaborating on a new or existing operational program, potential research partners should ask:

- What specific outcome is the program trying to achieve, and for which patients?
- What is the “conceptual model” for how the program will achieve these objectives?
- Will the program’s success improve quality or efficiency?
- How will this *collaboration* help the program succeed?

Sometimes the answers to these questions will not be clear up front. Working closely with clinical and operational leaders to frame and address these questions ultimately may be one of the most useful things a researcher can contribute.

## ASSESSING THE RESOURCE NEEDS

Once these stakeholder needs and program goals have been outlined, the next step is to work through what the research–operational partnership would require to help *answer* these questions, generate knowledge, and increase care value. This requires assessing the feasibility of the collaboration, and asking:

- What data are needed to measure program processes and outcomes?
- What resources are available to collect and analyze that data?
- What is the timeline for obtaining results?
- How will the findings be disseminated and implemented to change practice?

Often, there is strong mutual interest in collaboration, but the right data to answer the question of interest either are not available or are too expensive to acquire and analyze. Even if resources exist, there may be insufficient time to generate answers that inform operational decisions, or insufficient bandwidth to disseminate results and change practice. Note that parts of the project that are considered “research” to create generalizable knowledge may require institutional review board (IRB) oversight, which adds additional steps to the process. Helping leadership partners work through these issues is another important contribution researchers can make at this stage.

## ASSESSING THE RESEARCHER’S STRATEGIC GOALS

Researchers should remember that research to bridge the quality chasm is bidirectional: real-world innovations are often leading the way in care improvement,^1^ and researchers can gain valuable opportunities to add to their own knowledge by participating. As a researcher, it is important to understand how to strategically participate in operational programs in ways that best leverage your expertise and build your career.^2^ If you are a researcher, you should ask yourself:

- What can I learn by participating in this collaboration?
- What expertise and skills will I bring to this collaboration?
- How much of my time is required to participate, and how will that time be covered?
- How will this collaboration help me obtain future funding?
- How much interest is there in publishing the results?
- Will results be disseminated even if they are negative?

While understanding how a potential collaboration will help achieve career goals is important for all researchers, it is particularly relevant for early-stage investigators. Working with clinical and operational leaders is an exciting opportunity, but it is important to assess the right level and type of commitment for where you are in your career. This is where strong mentoring in delivery science methods and stakeholder engagement is critical for success.^2^

## ASSESSING LEADERSHIP STRATEGIC GOALS

Involving researchers in an operational or clinical program can be highly rewarding for health care system leaders. It can increase the chances that a program will generate robust and useful results, and there is potential that innovative work will be published. Melding research and operations is also a powerful way to improve health care value while enhancing careers and reputations. However, research often follows a slower time course than operational work, and engaging with researchers may slow the pace of typical “rapid improvement” processes. Leaders may also need to reach out to potential research collaborators within delivery system research divisions, universities, or clinical departments to initiate these relationships as well. If you are a clinical or operational leadership partner, you should ask yourself:

- Are there potential research collaborators I can partner with on this project?
- How can this program innovation improve its assessment of value and impact?
- Does the timeline for research collaboration fit with the timeline for implementation?
- How will this collaboration help me with future operational improvement efforts?
- Does this project require a level of rigor beyond the usual approaches to performance improvement?

There are no “right” answers to any of the questions posed above. Working through this checklist is a process that can help build a relationship based on trust and common principles that will probably take more than one email or meeting (and perhaps some “soul searching” as well) to complete. This checklist is designed to help shape the approach to collaborating, and to decide how to proceed in a way that builds learning health care systems 5, generates usable evidence that can be translated into action, and ultimately improves the quality and value of patient care.
